# A Comparative Study of the Chemical Properties and Antibacterial Activity of Four Different Ozonated Oils for Veterinary Purposes

**DOI:** 10.3390/vetsci11040161

**Published:** 2024-04-01

**Authors:** Gabrielė Slavinskienė, Aidas Grigonis, Marija Ivaškienė, Ingrida Sinkevičienė, Vaida Andrulevičiūtė, Liudas Ivanauskas, Dalia Juodžentė, Kristina Ramanauskienė, Gintaras Daunoras

**Affiliations:** 1 Dr. L. Kriaučeliūnas Small Animal Clinic, Veterinary Faculty, Lithuanian University of Health Sciences, LT-47181 Kaunas, Lithuania; aidas.grigonis@lsmu.lt (A.G.); marija.ivaskiene@lsmu.lt (M.I.); dalia.juodzente@lsmu.lt (D.J.); 2 Department of Biochemistry, Faculty of Medicine, Lithuanian University of Health Sciences, LT-47181 Kaunas, Lithuania; ingrida.sinkeviciene@lsmu.lt (I.S.); vaida.andruleviciute@lsmu.lt (V.A.); 3 Department of Analytical and Toxicological Chemistry, Faculty of Pharmacy, Lithuanian University of Health Sciences, LT-47181 Kaunas, Lithuania; liudas.ivanauskas@lsmu.lt; 4 Department of Clinical Pharmacy, Faculty of Pharmacy, Lithuanian University of Health Sciences, LT-47181 Kaunas, Lithuania; kristina.ramanauskiene@lsmu.lt

**Keywords:** ozonated linseed, hemp seed, sunflower and olive oils, chemical parameters, antibacterial activity

## Abstract

**Simple Summary:**

Currently, animal skin infections are treated with antimicrobial drugs. However, due to developing resistance, alternatives are being sought. Such an alternative to these drugs could be ozonated oils, exercising antibacterial and antifungal properties. This article presents data evaluating the chemical parameters and antibacterial activity of four selected ozonated oils (linseed, hemp seed, sunflower, and olive). The ozonation of oils changed their chemical composition. This was related to antibacterial activity, as the results showed a tendency for the reference strains of *S. aureus*, *E. faecalis*, and *E. coli* to have broader zones of inhibition (*p* < 0.001) than clinical strains. Overall, ozonated linseed oil exhibited the highest antibacterial activity, while ozonated olive oil showed the lowest. Further characterisation of selected ozonated oils will be performed to determine antifungal properties and cytotoxicity together with evaluation of shelf life of biological activity, prior to preclinical animal studies.

**Abstract:**

Infectious skin diseases are quite common in veterinary medicine. These diseases can be caused by both bacteria and pathogenic fungi. Antimicrobial drugs are usually used for treatment. An alternative to these drugs could be ozonated oils with antibacterial and antifungal properties. Four different ozonated oils (linseed, hemp seed, sunflower, and olive) were tested in order to develop an optimal pharmaceutical form for the treatment of skin infections in animals. Chemical parameters such as acid and acidity value, iodine and peroxide value, viscosity, and infrared spectres were analysed. The ozonation of oils resulted in changes in their chemical composition. The antimicrobial activity of the tested oils was evaluated by determining the minimum inhibitory concentrations and zones of inhibition in agar. After ozonation, the acid content increased in all the tested oils. The highest acidity was found in linseed oil (13.00 ± 0.11 mg KOH/g; 6.1%). Hemp oil, whose acidity was also significant (second only to linseed oil), was the least acidified by ozonation (11.45 ± 0.09 mg KOH/g; 5.75%). After ozonation, the iodine value in oils was significantly reduced (45–93%), and the highest amounts of iodine value remained in linseed (47.50 ± 11.94 g Iodine/100 g oil) and hemp (44.77 ± 1.41 Iodine/100 g oil) oils. The highest number of peroxides after the ozonation of oils was found in sunflower oil (382 ± 9.8 meqO_2_/kg). It was found that ozonated hemp and linseed oils do not solidify and remain in liquid form when the temperature drops. The results showed a tendency for the reference strains of *S. aureus*, *E. faecalis*, and *E. coli* to have broader zones of inhibition (*p* < 0.001) than clinical strains. Overall, ozonated linseed oil had the highest antibacterial activity, and ozonated olive oil had the lowest, as determined by both methods. It was found that ozonated linseed oil was the most effective on bacteria, while the most sensitive were *S. aureus* ATCC 25923, MRSA, and *S. pseudointermedius* (MIC 13.5 mg/mL, 4.6 mg/mL, and 13.5 mg/mL, respectively, and sterile zones 20.67 ± 0.98 mm, 20.25 ± 0.45 mm, and 18.25 ± 0.45 mm, respectively). The aim and new aspect of this work is the characterisation of selected ozonated vegetable oils, especially hemp oil, according to chemical and antibacterial parameters, in order to select suitable candidates for preclinical and clinical animal studies in the treatment of bacterial or fungal skin infections in terms of safety and efficacy.

## 1. Introduction

In clinical veterinary medicine, animal skin infections are one of the most common pathologies. Depending on the animal species, they include folliculitis; furunculosis; abscesses; superficial pyoderma; superficial, pustular, and crusting dermatitis; impetigo; otitis externa; infected wounds; mastitis; and interdigital necrobacillosis and if not controlled can lead to life-threatening septicaemia [1,2,3,4]. Dermal injuries can be colonised by aerobic and anaerobic bacteria; more detailed data related to the most frequent pathogens causing animal skin infections are listed in Table 1.

Bacterial skin infections can be treated with topical medications (creams, ointments, shampoos, lotions, mousses, sprays, and wipes containing antiseptics or antibiotics) or systemic antibiotic therapy [4]. Reducing the pressure of bacterial infections through therapeutic means not only improves animal health but also positively affects animal welfare and human safety. However, bacteria responsible for skin infections in animals have exhibited significant resistance to antibiotics, emphasising the need for heightened awareness, the rational utilisation of these medications, and research of alternatives [5,6,7,8,9,10]. The use of bacteriophages, bacteriocins, antimicrobial photodynamic therapy, phytochemicals, and ozone have recently been investigated as alternative treatment options [11]. Among the alternatives, research on ozonated oils has been increasing in recent years. It is worth noting that, compared to ozone gas and ozonated water, ozonated oils have better stability and ease of handling, have better storage, avoid rapid degradation, allow for treatment outside the hospital, and reduce the risk of using large and inappropriate doses [12].

**Table 1:** The pathogenic bacteria related to animal skin infections [compiled from [1,2,3,4,8,10]].

Animal Species	Bacterial Pathogens
Cattle	*Actinomyces bovis*, *Bacteroide melaninogenicus*, *Staphylococcus aureus*, *Streptococcus dysgalactiae*, *Fusobacterium necroforum*, *Moraxella bovis*, *Trueperella pyogenes*
Pigs	*Dermatophylus congolensis*, *S. hyicus*, *S. intermedius*, *S. chromogenes*, *S. sciuri*
Goats	*Dermatophylus congolensis*, *S. aureus*, *S. hyicus*, *S. haemolyticus*, *S. warneri*, *S. epidermidis*, *S. chromogenes*, *S. caprae*, *S. simulans*
Sheep	*Dermatophylus congolensis*, *Corynebacterium pseudotuberculosis*, *Pithomyces fungus*, *S. aureus*, *S. xylosus*, *S. epidermidis*, *Str. Dysgalactiae*
Poultry	*S. aureus*, *S. hyicus*
Dogs	*Staphylococcus pseudintermedius*, *Pseudomonas aeruginosa*, coagulase-negative *Staphylococcus* (CoNS) (*S. xylosus*, *S. simulans*, *S. epidermidis*, *S. sciuri*, *S. chromogenes*, *S. hyicus*, and *S. cohnii*), *Streptococcus* (*S. canis*, *S. mitis*, *S. dysgalactiae*, *S. agalactiae*), *E. coli* and *Enterobacterales* (*Klebsiella pneumoniae*, *Proteus mirabilis*, *Raoultella ornithinolytica*, *Enterobacter cloacae*, *Serratia marcescens*, and *Citrobacter youngae*), *E. faecalis*
Cats	coagulase-negative *Staphylococcus*, *S. aureus*, *S. pseudintermedius*, *Streptococcus canis*, *Pseudomonas aeruginosa*, *E. coli* and *Klebsiella pneumoniae*

Several in vitro studies have evaluated the antimicrobial activity of ozonated oils. Bouzid et al. found that ozonated olive oil, depending on the concentration used (0.248 to 63.5 mg/mL), effectively inhibited the growth of *Proteus mirabilis* ATCC 35659, *Escherichia coli* ATCC 25922, and *Staphylococcus aureus* ATCC 6538; however, the highest ozone concentration was required to inhibit the growth of *Listeria monocytogenes* ATCC 15313, *Pseudomonas aeruginosa* ATCC 27853, and *Klebsiella pneumoniae* ATCC 4352 [13]. In addition, several studies have shown that many oils with varying levels of ozonation have antibacterial, fungicidal, and antiviral properties [14,15,16,17,18,19,20]. Consequently, ozonated oils have been successfully used in humans to treat various skin conditions and sensitivities such as atopic dermatitis; contact dermatitis; ichthyosis; psoriasis; acne; abrasions; pressure ulcers; insect bite blisters; first-degree burns; diabetic foot; and skin care after laser therapy, surgery, and sunburn [15]. The therapeutic activity of ozonated oil was attributed to its antimicrobial, antihypoxic, analgesic, and immunomodulatory effects [21,22]. The efficacy and safety of ozonated oils for veterinary purposes have also been tested. Ozonated olive oil was found to be effective in *in vivo* studies in rats against *S. pyogenes* and *S. aureus* [23]. The topical application of ozonated vegetable oil in MRSA-infected rat skin showed a slight reduction in bacteria load and improved wound healing [24]. Ozonated olive oil has been used effectively to treat cat ear mites (*Otodectes cynotis*) [25]. The ozonated oil ointment used in the study by Szponder T. et al. proved to be an effective and convenient means for the topical treatment of foot rot in sheep enabling precise administration and causing no undesirable effects [26]. Ozonated oils in liposomes plus hypromellose have been reported to have restorative and regenerative effects in external spontaneous ocular pathologies in animals and humans [27]. The successful use of ozonated oil in the treatment of pharmacodermia in female dogs has been reported [28].

In order to effectively use ozonated oils for the treatment of animal skin infections, it is necessary to study the parameters that could potentially influence their therapeutic effectiveness. Chemical tests of peroxide, iodine and acidity, viscosity, and spectroscopy (FT-IR, 1H NMR, and 13C NMR) [29], together with antimicrobial tests, have been performed to characterise the prepared ozonated oils [30,31]. It is observed that ozonated oil with a higher peroxide value has better antimicrobial activity [16,30,32]. The amount of peroxide in ozonated vegetable oils can be increased by adding water and increasing the ozonation time [16]. Two studies showed an increase in antimicrobial activity with ozonation times up to 5 h, after which antibacterial activity did not improve [32,33]. Different ozonation techniques, chemical parameters before and after ozonation, and their correlation with antimicrobial activity within single oil have been analysed in several studies with interesting observations [29,31,32,34,35].

However, Guerra-Blanco et al. confirmed that the ozonation kinetics of different oils (grapeseed, sunflower, avocado, and olive) differ [36]. Therefore, such an analysis of several different oils would help to gain a broader picture of the characteristics of ozonated oils and to find an optimal formulation suitable for the treatment of skin infection in animals that would be an alternative to the use of antibiotics and the prevention of antibiotic resistance. This study analyses the chemical parameters of linseed, hemp seed, sunflower, and olive oil before and after ozonation and the correlation between these parameters and the antimicrobial activity of the studied preparations.

As the first phase of the investigation, this study aimed to evaluate the chemical parameters (acid and acidity values, iodine values and peroxide values, viscosity, and infrared spectres) and antimicrobial activity of the selected ozonated oils (linseed, hemp seed, sunflower, and olive oil) in order to develop an optimal pharmaceutical form for the treatment of skin infections in animals.

## 2. Materials and Methods

### 2.1. Materials

For antimicrobial assays, Mueller–Hinton agar media (Sigma-Aldrich, Poznan, Poland) and LB broth (Miller) (Sigma-Aldrich, Poznan, Poland) were used. All reagents for chemical analysis were purchased from Sigma-Aldrich: sodium thiosulfate anhydrous, hexane, glacial acetic acid, chloroform, methanol, potassium iodide, and potassium hydroxide. The ozonated oils used in this study (linseed, hemp seed, sunflower, and olive oil) were specifically prepared for research purposes and were generously provided by UAB Ozono Centras, Lithuania. Oils were ozonated for 4 h without water, and the ozonation time was chosen after analysing the results of other scientists’ research [30,36,37]. This was the minimum time during which good antimicrobial activity results are usually obtained. All used oils were extra virgin and cold-pressed.

### 2.2. Bacterial Strains

Four ozonated oils were tested against Gram-positive *S. aureus* (ATCC 25923 and a clinical strain), the MRSA clinical strain, *S. pseudintermedius* clinical strain, *E. faecalis* (ATCC 29212 and a clinical strain), Gram-negative *P. aeruginosa* (ATCC 27859 and a clinical strain) and *E. coli* (ATCC 25922 and a clinical strain). The clinical strains were obtained from the collection of the Institute of Microbiology and Virology (Faculty of Veterinary Medicine, Lithuanian University of Health Sciences).

### 2.3. FT-IR Analysis

FT-IR spectra of hemp seed, linseed, sunflower, and olive oil before and after ozonation were recorded by using a Shimadzu IRTracer-100 (Kyoto, Japan) FT-IR spectrophotometer. Transmission levels were measured for wave numbers of 4000–400 cm^−1^ with a resolution of 2 cm^−1^ for 45 scans and read as absorbance in triplicate before taking the averaged value. About 2 μL of the sample was deposited between two disks of KBr, avoiding air bubble formation.

### 2.4. Peroxide Value

The peroxide value (PV) is a measure of the amount of oxygen chemically bound to an oil or fat as peroxides, particularly hydroperoxides. It is one of the indicators of the strength of the ozonation process. PV is expressed in milliequivalents of active oxygen per kilogram of oil (meq/kg). PV was determined using the ISO standard 3960:2017 [38].

### 2.5. Acid Value and Acidity

The acid value is the number of milligrams of potassium hydroxide required to neutralise the free fatty acids present in 1 g of fat when determined in accordance with the procedure specified in LST EN ISO 660:2020 [39]. The acid value is expressed in milligrams per gram.

Acidity is the content of free fatty acids, also determined according to the procedure specified in LST EN ISO 660:2020 [39]. The acidity is expressed as a percentage by mass. If the result of the determination is reported as acidity without further explanation, this is, by convention, the acidity based on the oleic acid content.

### 2.6. Iodine Value

The iodine value (IV) is a mass of halogen, expressed as iodine, absorbed by the test portion following the specified procedure by ISO 3961:2018 [40], divided by the mass of the test portion. The IV is expressed as a mass fraction in grams per 100 grams of fat.

### 2.7. Viscosity

The dynamic viscosity of ozonated the oil samples was measured in an ANTON PAAR modular compact rheometer MCR-102 (Anton Paar GmbH, Graz, Austria), at different shear rates and three controlled temperatures (5, 22, and 38 ± 0.1 °C). The shear rate in the range from 0.1 to 100 s^−1^ was tested. The measurement was performed in a parallel plate system with a diameter of 50 mm at a gap of 0.2 mm. Data were processed using the Rheocompass software (ver. 1.14).

### 2.8. Minimum Inhibitory Concentration

The minimum inhibitory concentration (MIC) was determined using the broth microdilution method. The assay was carried out in 96-well microtitre plates filled with an LB medium. Ozonated oils were transferred to microplate wells in order to obtain two-fold serial dilution in concentrations ranging between 0.8 and 310 mg/mL. The final concentrations of bacteria in the wells were 1–3 × 10^5^ CFU/mL. The plates were incubated at 37 °C for 24 h. There were three types of controls: negative control (only medium), positive control (medium and bacteria inoculum), and colour control (medium and ozonated oils in serial dilutions). Bacterial growth was evaluated with a microplate photometer Multiskan FC (Thermo Scientific, Foster city, CA, USA) in 570 nm wavelength. Based on the control samples, thresholds were defined to determine the criteria for classifying the obtained results as either bacterial growth or non-growth.

### 2.9. Agar Well Diffusion Method

The inoculum suspensions of the bacterial isolates were adjusted to 1.0 × 10^5^ CFU/mL in sterile saline. Plates (90 mm diameter) with Mueller–Hinton agar were streaked evenly in three directions over the entire agar surface with a swab dipped into the bacterial inoculum suspension. The plates were allowed to dry before making 9 mm wells with a sterile metal cylinder. Wells were filled with control oils (not ozonated) and ozonated oils. The plates were incubated at 37 °C for 24 h. After incubation, the inhibition zones were measured.

### 2.10. Statistical Analysis

For agar well diffusion assay, the results are expressed as the mean ± SD of at least 12 independent measurements. The broth microdilution assay was repeated until consistent MIC results were obtained for each bacterial strain. Analysis of variance (ANOVA) was employed to compare the results between all four ozonated oils. Student’s *t*-test was used to compare the means between two groups (e.g., inhibition zones between clinical and reference strains). The correlations were determined using the Pearson test after verifying the normality using the Shapiro–Wilk test. For statistical calculations involving agar well diffusion data where the inhibition zone was not observed, a well diameter of 9 mm was employed in the analysis. Statistical analyses were applied to the data using the SPSS statistical package (IBM SPSS Statistics for Windows 29.0).

## 3. Results

### 3.1. Acid Value (AV) Results

An increase in the acid value (AV) after ozonation was observed in all four oils tested (Table 2). The highest increase was in linseed oil—18 times. It also had the highest acid value after ozonation, at 13 mg KOH/g. The lowest increase in the acid value after ozonation was observed in hemp seed oil (2.8 times), but it still had a relatively high acid value of 11.45 mg KOH/g. The lowest acid value after ozonation was in olive oil, at 3.65 mg KOH/g. As expected, acidity results were consistent with acid values of the same oils (Table 3).

### 3.2. Iodine Value (IV) Results

The iodine values before ozonation were compatible with ordinary values that were defined by other researchers [15,37], where linseed had the highest IV, and olive oil had the lowest. During the ozonation reaction, a decrease in iodine values was observed (Table 4). The iodine value difference before and after ozonation in all oils was relatively similar—on average 42.83 g/100 g oil (39.34–47.75). This could be due to the same ozonation time (4 h).

### 3.3. Peroxide Value (PV) Results

The peroxide value increased in all tested oils after ozonation (Table 5). Sunflower oil had the highest peroxide value after ozonation. The other three oils had very similar peroxide values.

### 3.4. Viscosity

Viscosity results are shown in Table 6. A strong negative linear correlation was found: Lower temperatures showed higher consistency factor (K) measures of ozonated oils (r = −0.953–−0.881, *p* > 0.05). We additionally observed that ozonated sunflower oil (OSO) and ozonated olive oil (OOO) solidified in the refrigerator temperature (5 °C), but ozonated hemp seed oil (OHO) and ozonated linseed oil (OLO) remained liquid. OOO at 5 °C was the most viscous of all tested oils and the least viscous at 38 °C, with a 1712× difference between those two values. Such a change in the consistency factor is not characteristic of unprocessed olive oil. It is rather an impact of ozonation. The flow behaviour index characterised the ozonated oils as pseudoplastic fluids.

### 3.5. Fourier-Transform Infrared Spectroscopy (FT-IR)

**Figure 1:**
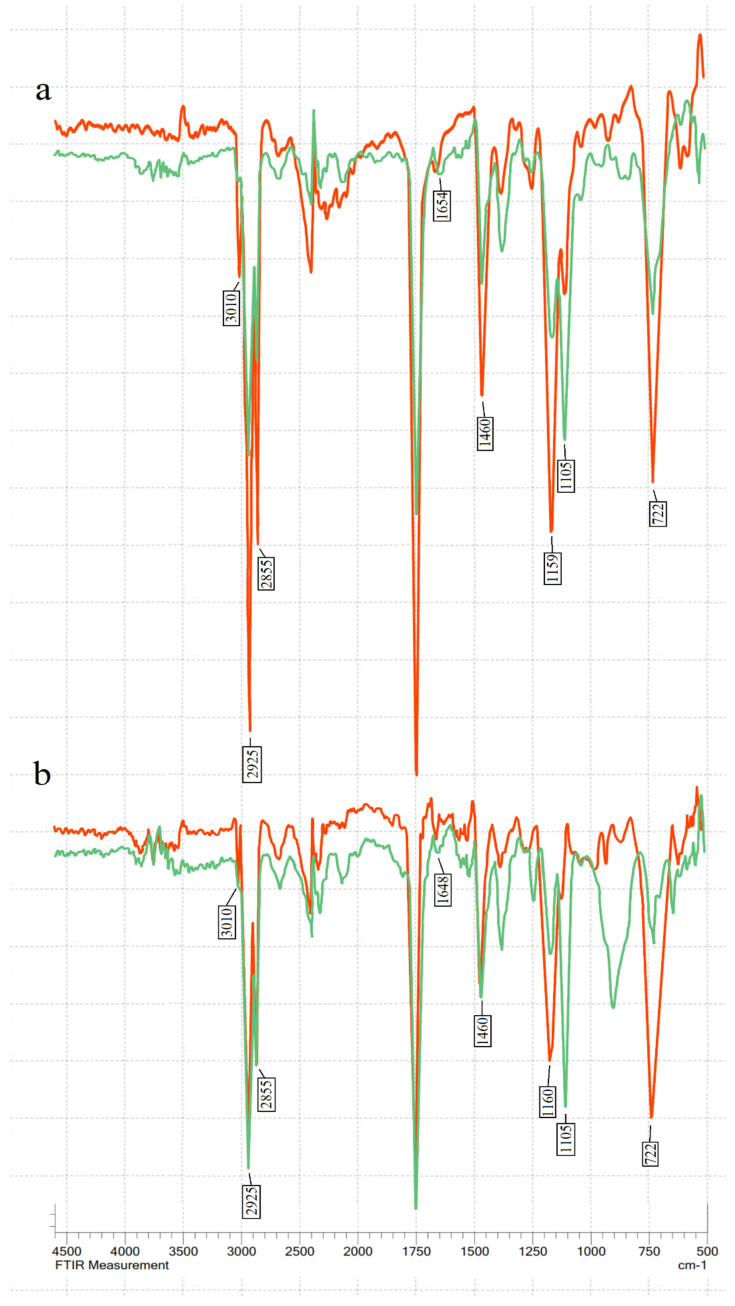

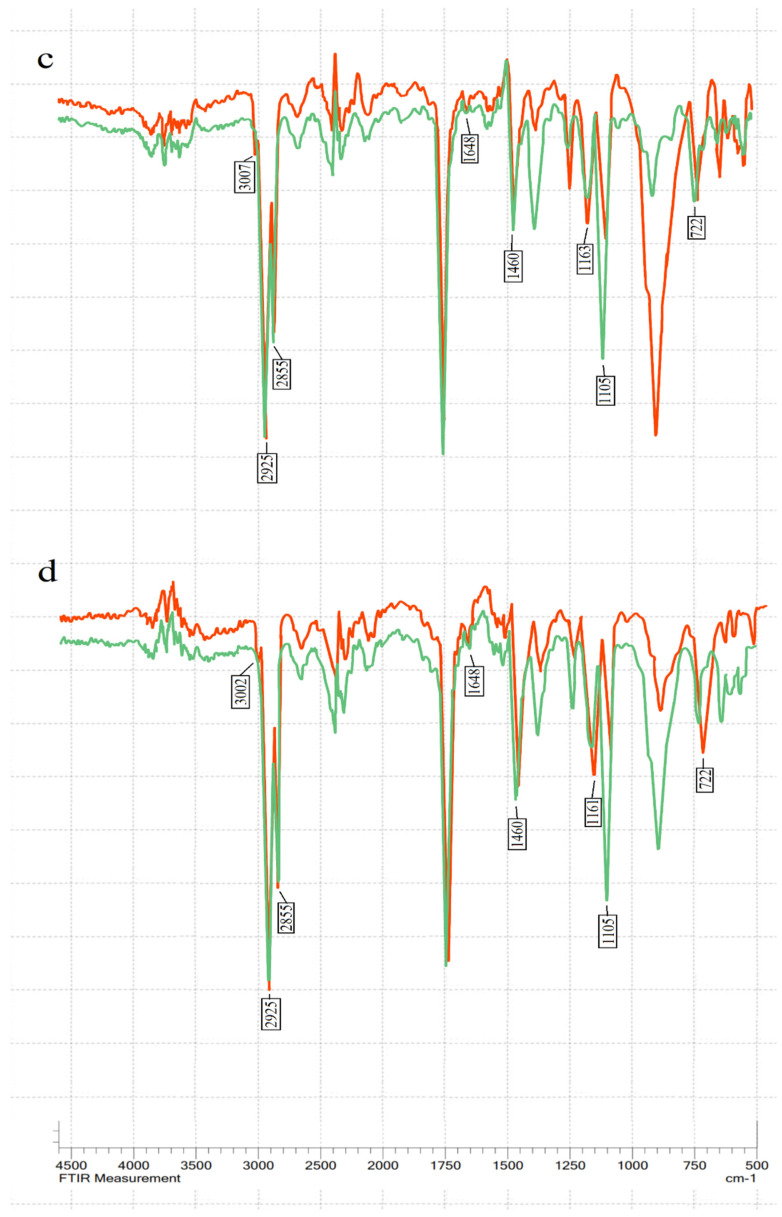
FT-IR spectra of oils before (red line) and after (green line) ozonation: (**a**) linseed oil; (**b**) hemp seed oil; (**c**) sunflower oil; (**d**) olive oil.

The transmittance % was measured in the mid-IR spectrum range (400–4000 cm^−1^) (see Figure 1). The main changes during ozonation occurred at 722–723 cm^−1^ (overlapping of the methylene rocking vibration and the out-of-plane vibration of cis-disubstituted olefins), 1105 cm^−1^ (–C–O stretching), 1155–1163 cm^−1^ (sv of –C–O), 1460–1465 cm^−1^ (the bending vibrations of CH_2_ and CH_3_ aliphatic groups), 1654 cm^−1^ (the C=C stretching vibration of cis-olefins), 3009 cm^−1^ (C=C–H stretching), and in the bands at 2922 and 2853 cm^−1^, which are attributed to the symmetric and asymmetric stretching vibration of the aliphatic CH_2_ group [41,42,43,44].

The characteristic double-bond absorption bands at 722 cm^−1^, 1648–1654 cm^−1^, and 3009 cm^−1^ decreased after ozonation in all four oils, showing double-bond consumption. A decrease in absorption bands 1155–1163 cm^−1^ and 1460–1461 cm^−1^ was also visible. The peaks at 2925 and 2855 cm^−1^ decreased after ozonation in all tested oils. Meanwhile, the peak at C–O stretching in 1105 cm^−1^ increased after ozonation. This indicates ozonide formation.

There were no visible peaks between 3300 and 3600 cm^−1^, indicating the absence of hydroxyl groups in the tested ozonated oils [17]. In addition, no aldehydes were detected in FT-IR analysis after ozonation (no peaks were observed in aldehyde characteristic absorption bands between 2700 and 2800 cm^−1^ [34].

### 3.6. Minimal Inhibitory Concentrations

Gram-positive *Staphylococcus aureus* ATCC 25923 was the least resistant of the tested microorganisms (Table 7), which corresponds to other authors’ results [31,33]. OOO had the highest MIC between 190.5 and 300 mg/mL for all the microorganisms tested and was the least effective. The MIC values for OHO ranged from 18.5 to 74 mg/mL, and for the tested isolates from OSO, they ranged from 4.7 to 80 mg/mL. OLO showed the lowest MIC values against the tested microorganisms between 4.6 and 50 mg/mL, except for *S. aureus* ATCC 25923, where the MIC of OSO was lower than that of OLO. The MIC results had a strong negative correlation with the acid values of the same ozonated oils (R = −0.920, *p* < 0.001). The ozonated oils with higher acid values had lower MIC values (better antimicrobial activity). It was also observed that the higher the percentage of oil iodine consumption after ozonation, the higher the minimum inhibitory concentrations (R = 0.971, *p* = 0.029). OOO had the highest MICs for all the tested microorganisms in the range from 190.5 to 300 mg/mL and was the least effective.

The control samples of oils before ozonation had no antibacterial activity. The MIC values for OHO against the tested isolates ranged from 18.5 to 74 mg/mL, and for OSO, they were in the range from 4.7 to 80 mg/mL. OLO showed the lowest MIC values against the tested microorganisms, ranging from 4.6 to 50 mg/mL, except for *S. aureus* ATCC 25923, where OSO had a lower MIC than OLO. MIC results had a strong negative correlation with acid values of the same ozonated oils (R = −0.920, *p* < 0.001). The ozonated oils with higher acid values had lower MIC values (better antimicrobial activity). It was also observed that the higher the percentage of iodine consumption of oil after ozonation, the higher the minimum inhibitory concentrations (R = 0.971, *p* = 0.029). MIC results had no correlation with peroxide values (R = −0.215, *p* > 0.05). The MIC assay with *E. faecalis* reference and clinical strains was not performed. In relation to the agar well diffusion method, this pathogen tends to require higher ozonated oil concentration, especially for the tested olive oil.

### 3.7. Agar Well Diffusion

The results showed that the reference strains of *S. aureus*, *E. faecalis*, and *E. coli* had wider zones of inhibition (*p* < 0.001) than clinical strains. There were some exceptions where no statistically significant difference between the two strains was observed for each bacterium: *E. faecalis* with OLO, *S. aureus* with OOO, and *E. coli* with OSO. The *P. aeruginosa* reference strain had smaller zones of inhibition than the clinical strain (*p* < 0.001 with OHO and OSO, *p* < 0.01 with OLO).

The antimicrobial efficacy of the ozonated oils in Gram-group bacteria was not clearly demonstrated. The difference between the diameters of the inhibition zones was more pronounced between two Gram-negative bacteria (*E. coli* and *P. aeruginosa*) than between different groups of Gram-negative bacteria. Control samples of oils before ozonation did not show zones of bacterial inhibition. The correlation between the two methods showed that the sensitivity of the bacteria to a given oil was consistent. Ozonated oils that produced lower MICs also produced wider zones of inhibition with the same bacteria (Table 8). Overall, OLO showed the highest antibacterial activity, and OOO showed the lowest, as observed by both methods. The strength and significance of this correlation varied slightly between the different bacteria. Both reference and clinical *E. coli* and *P. aeruginosa* strains had a statistically significant strong correlation (r = −0.981, *p* < 0.05, r = −0.970, *p* < 0.01, r = −0.978, respectively, *p* < 0.001). The results of MIC had a strong negative, but statistically non-significant, correlation with the results of agar well diffusion assay in *S. pseudintermedius* (r = −0.930, *p* = 0.070) and *MRSA* (r = −0.910, *p* = 0.090) for the same ozonated oils. The correlation between the two assays of *S. aureus* ATCC 25923, clinical *S. aureus*, and *P. aeruginosa* ATCC 27859 was weaker and more non-significant (r = −0.707, *p* = 0.293; r = −0.711, *p* = 0.011 and r = −0.652, *p* = 0.011).

## 4. Discussion

In order to standardise the ozonation process, many authors suggest monitoring the peroxide value and viscosity parameters during the process [18]. The values of peroxides obtained in our study are among the lower ones. This was due to the choice not to add water during the process and the selected ozonation time (4 h). The research of Moureu et al. clearly shows that the ozonation of oil with water leads to higher peroxide values (up to 2600 mEq of active oxygen/kg oil with water and 430 without) [33]. In addition, it should be emphasised that peroxide values can be highly dependent on the chosen peroxide determination method [29]. When tracking viscosity, it is important to pay attention to temperature. As our study shows, the viscosity of ozonated oils varies greatly at different temperatures. Unfortunately, some studies with ozonated oils do not specify the temperature for the viscosity parameter. Obtaining ozonated oil with precisely matched parameters by repeated tests remains a challenging task.

According to many authors, the peroxide content is the most important characteristic of the antimicrobial efficacy of ozonated oils [16,30]. In most cases, increasing levels of peroxide have better antimicrobial activity. In our research, the peroxide value had no correlation with antimicrobial activity. This may be due to relatively similar PV concentrations.

In this study, the highest PV (OSO) was only 72% higher than the lowest PV (OLO). It is also possible that there is a stronger correlation between the peroxide value and antimicrobial activity when comparing these parameters in the same oil than in different oils, as shown in our study. However, our antimicrobial results were correlated with the acid value and mole percentage of double bonds consumed. Moureu et al. observed that the acid value also correlated with antimicrobial activity [33]. In addition, Guerra-Blanco et al. observed that anti-inflammatory and wound-healing effects were achieved with ozonated grape seed and sunflower oil with a low degree of ozonation, implying that the superior therapeutic effect of ozonated oils is not necessarily due to a high peroxide value [36].

Diaz et al. observed that the oil with the lowest iodine index (highest oleic acid content) reacted faster with ozone, consuming 94 percent of the moles of double bonds, while other oils consumed 50–60% at the same time [31]. Our study supports this observation, with olive oil having the lowest iodine index before ozonation and the highest consumption of double bonds (93%) compared to other oils (45–60%). Diaz et al. (2021) analysed this phenomenon in more detail and discussed the possible reasons. It is worth noting that our oil with the highest consumption of double bonds (olive oil) and the one in the study by Diaz et al. (“Dende” oil) yielded opposite results regarding their PV and antimicrobial efficacy. Despite the similar IV pre-ozonation and double-bond rate, Dende oil had the strongest antimicrobial activity among the oils with the highest peroxide value, while our ozonated olive oil had the weakest antibacterial activity. These observations suggest that the ability of oils to absorb ozone is not the main determinant of their potential antimicrobial activity [18].

The main changes in the FT-IR spectrum after ozonation were consistent with earlier studies [24,35,37]. During FT-IR analysis, it was observed that different oils had slightly different frequency numbers for the same chemical groups, especially for the 3006 cm^−1^ band. Hemp seed and linseed oil had a peak at 3009.97 cm^−1^, sunflower at 3007 cm^−1^, and olive oil at 3002 cm^−1^.

The exact position of this band depends on the proportion of fatty acids [17]. Oils with higher unsaturation have a higher frequency [19]. After ozonation, the oils in our study had no deformations in the characteristic bands of aldehyde and hydroxyl. This was an expected result since ozonation did not use water. Ozonolysis can produce aldehydes and α-hydroxy hydroperoxides if a protic solvent (water) is present. However, FT-IR results do not necessarily mean that the sample is free of aldehydes. Kogawa et al. and Soriano et al. showed that ozonated oils contained a small amount of aldehyde when analysed using H NMR, even though FT-IR analysis showed no peaks in the characteristic aldehyde band [34,37].

Our study did not observe any evidence that Gram-positive or Gram-negative bacteria were more sensitive to ozonated oils compared to each other. Our results did not fully agree with other studies by different authors, which reported that Gram-positive bacteria are more sensitive to ozonated oils [31,45]. Although there are frequent references to the fact that Gram-positive bacteria are more sensitive to ozonated oils than Gram-negative bacteria, there is still a lack of comprehensive articles on this topic based on a larger number of representatives of both groups and providing stronger statistical support.

We examined the correlation between the two antimicrobial activity assay methods and found that the correlation was not significant for certain bacteria. It can be assumed that this non-significance is due to the identical MIC values observed with the different ozonated oils. However, in reality, the true MIC value can be anywhere between the last dilution that inhibits growth and the first dilution that does not inhibit growth [20]. Therefore, the exact values could have been different, leading to different levels of significance. In addition, some correlations were observed to be non-significant due to OSO having a lower minimum inhibitory concentration (MIC) than OLO against *S. aureus* ATCC 25923 or a larger zone of inhibition than OHO against *S. pseudintermedius*. The presence of such deviations can be attributed to the relatively higher peroxide value of sunflower oil compared to the other three oils. However, we cannot assume that certain bacteria are more sensitive to OSO, since the second method showed that OLO remained the most effective.

Promising results were obtained with the tested ozonated oils, allowing for the development of an effective and safe antimicrobial/antiseptic pharmaceutical formulation for external use against skin infections, which can be used as an alternative (or adjunctive) to veterinary antibiotics. A more extensive in vitro evaluation of the bioactivity of the selected ozonated oils is necessary before preclinical animal studies. Therefore, we plan to carry out an assessment of the antifungal properties and cytotoxicity of ozonated linseed, hemp, sunflower, and olive oils in cell lines and the shelf life of biological activity.

## 5. Conclusions

Ozone formulation and therapy are gaining popularity in research and clinical settings [14,46,47] as well as in veterinary medicine [48]. Four different ozonated oils (linseed, hemp, sunflower, and olive) were tested in order to develop an optimal pharmaceutical form for the treatment of skin infections in animals. The analysed chemical parameters were acids and acidity, iodine and peroxide content, viscosity, and infrared spectra. The ozonation of oils changed their chemical composition. The antimicrobial activity of the tested oils was evaluated by determining the minimum inhibitory concentrations and zones of inhibition in agar. The results showed that the reference strains of *S. aureus*, *E. faecalis*, and *E. coli* had wider zones of inhibition (*p* < 0.001) than clinical strains. Overall, ozonated linseed oil had the highest antibacterial activity, and ozonated olive oil had the least, as determined by both methods. Further characterisation of the selected ozonated linseed, hemp, sunflower, and olive oils is necessary to determine antifungal properties and cytotoxicity together with the evaluation of the shelf life of biological activity, prior to preclinical animal studies.

## Figures and Tables

**Table 2:** Acid values before and after ozonation, mean ± SD.

Ozonated Oil	Before Ozonation, mg KOH/g	After Ozonation, mg KOH/g
Linseed	0.72 ± 0.01	13.00 ± 0.11
Hemp seed	4.10 ± 0.02	11.45 ± 0.09
Sunflower	2.20 ± 0.08	8.90 ± 0.02
Olive	0.25 ± 0	3.65 ± 0.03

**Table 3:** Acidity before and after ozonation, in %.

Oil	Before Ozonation	After Ozonation
Linseed	0.36	6.51
Hemp seed	2.06	5.75
Sunflower	1.11	4.47
Olive	0.25	1.84

**Table 4:** Iodine value before and after ozonation, mean ± SD.

g Iodine/100 g Oil	Linseed Oil	Hemp Seed Oil	Sunflower Oil	Olive Oil
Before ozonation	86.84 ± 5.98	84.42 ± 3.32	74.08 ± 0	51.11 ± 0.36
After ozonation	47.50 ± 11.94	44.77 ± 1.41	29.50 ± 1.21	3.36 ± 2.43
Consumption	39.34	39.65	44.58	47.75
Consumption in %	45	47	60	93

**Table 5:** Peroxide values, meqO_2_/kg ± SD.

	Linseed Oil	Hemp Seed Oil	Sunflower Oil	Olive Oil
Before ozonation	1 ± 0	13 ± 0	18 ± 2	0 ± 0
After ozonation	222.5 ± 12.4	247 ± 4.1	382 ± 9.8	240.5 ± 3.7

**Table 6:** Consistency factor, mPa·s.

	38 °C	22 °C	5 °C
OLO	1011.1	2968.8	12,375.0
OHO	507.04	1326.7	4475.6
OSO	491.93	1546.5	11,129.0
OOO	132.12	3325.2	226,270.0

**Table 7:** MICs of ozonated oils against tested bacteria, mg/mL.

	OLO	OHO	OSO	OOO
*S. aureus*	25	25	50	280
*S. aureus* ATCC 25923	13.5	18.5	4.7	190.5
*MRSA*	4.6	37.0	37.0	280.0
*S. pseudintermedius*	13.5	74.0	74.6	280.0
*E. coli*	50	60	67	300
*E. coli* ATCC 25922	35	35	80	280
*P. aeruginosa*	25	25	25	280
*P. aeruginosa* ATCC 27859	25	25	50	280

**Table 8:** Inhibition zone diameter in mm, mean ± SD.

	Linseed Oil	Hemp Seed Oil	Sunflower Oil	Olive Oil
*S. aureus*	20.0 ± 0	15.17 ± 0.39	13.42 ± 1.08	11.25 ± 0.45
*S. aureus* ATCC 25923	20.67 ± 0.98	16.0 ± 0	14.33 ± 0.98	11.33 ± 0.49
*MRSA*	20.25 ± 0.45	18.50 ± 0.52	15.50 ± 0.52	11.17 ± 0.39
*S. pseudintermedius*	18.25 ± 0.45	15.08 ± 0.67	16.17 ± 0.39	12.67 ± 0.49
*E. faecalis*	15.0 ± 0	n.a.	n.a.	n.a.
*E. faecalis* ATCC 29212	15.0 ± 0	13.42 ± 0.79	11.0 ± 0.43	n.a.
*E. coli*	18.50 ± 0.52	14.75 ± 0.62	19.58 ± 0.99	n.a.
*E. coli* ATCC 25922	21.58 ± 0.79	19.50 ± 0.52	20.42 ± 0.79	n.a.
*P. aeruginosa*	13.0 ± 0.43	12.0 ± 0	10.0 ± 0	n.a.
*P. aeruginosa* ATCC 27859 *	12.17 ± 0.83	11.75 ± 0.45	n.a.	n.a.

n.a. not active. Inhibition zone diameters differed statistically significantly between all four ozonated oils (*p* < 0.001). * Difference between means was statistically insignificant (*p* = 0.71) (Student’ *t*-test).

## Data Availability

The original data presented in the study are included in the present article; further inquiries can be directed to the corresponding author.
